# Guidelines for the management of neuroendocrine tumours by the Brazilian gastrointestinal tumour group

**DOI:** 10.3332/ecancer.2017.716

**Published:** 2017-01-26

**Authors:** Rachel P Riechelmann, Rui F Weschenfelder, Frederico P Costa, Aline Chaves Andrade, Alessandro Bersch Osvaldt, Ana Rosa P Quidute, Allan dos Santos, Ana Amélia O Hoff, Brenda Gumz, Carlos Buchpiguel, Bruno S Vilhena Pereira, Delmar Muniz Lourenço Junior, Duilio Reis da Rocha Filho, Eduardo Antunes Fonseca, Eduardo Linhares Riello Mello, Fabio Ferrari Makdissi, Fabio Luiz Waechter, Francisco Cesar Carnevale, George B Coura-Filho, Gustavo Andrade de Paulo, Gustavo Colagiovanni Girotto, João Evangelista Bezerra Neto, João Glasberg, Jose Claudio Casali-da-Rocha, Juliana Florinda M Rego, Luciana Rodrigues de Meirelles, Ludhmila Hajjar, Marcos Menezes, Marcello D Bronstein, Marcelo Tatit Sapienza, Maria Candida Barisson Villares Fragoso, Maria Adelaide Albergaria Pereira, Milton Barros, Nora Manoukian Forones, Paulo Cezar Galvão do Amaral, Raphael Salles Scortegagna de Medeiros, Raphael L C Araujo, Regis Otaviano França Bezerra, Renata D’Alpino Peixoto, Samuel Aguiar, Ulysses Ribeiro, Tulio Pfiffer, Paulo M Hoff, Anelisa K Coutinho

**Affiliations:** 1 Instituto do Câncer do Estado de São Paulo, Universidade de São Paulo, São Paulo 01246-000, Brasil; 2 Departamento de Radiologia e Oncologia da Faculdade de Medicina da Universidade de São Paulo, São Paulo 01246-903, Brasil; 3 Hospital Sírio-Libanês, São Paulo 01308-050, Brasil; 4 Hospital Moinhos de Vento de Porto Alegre, Porto Alegre 90035-000, Brasil; 5 Oncocentro, Belo Horizonte 30360-680, Brasil; 6 Departamento de Cirurgia, Universidade Federal do Rio Grande do Sul, Porto Alegre 90040-060, Brasil; 7 Hospital de Clinicas de Porto Alegre, Porto Alegre 90035-903, Brasil; 8 Departamento de Fisiologia e Farmacologia da Faculdade de Medicina da Universidade Federal do Ceará, Fortaleza 60020-180, Brasil; 9 Hospital Universitário Walter Cantidio, Ceará 60430-370, Brasil; 10 Instituto Nacional do Câncer, Rio de Janeiro 20230-240, Brasil; 11 Disciplina de Endocrinologia e Metabologia, Faculdade de Medicina da Universidade de São Paulo, São Paulo 01246-903, Brasil; 12 Department of Surgery, AC Camargo Cancer Centre, São Paulo 01509-010, Brasil; 13 Departamento de Gastroenterologia da Faculdade de Medicina da Universidade de São Paulo, São Paulo, Brasil; 14 Universidade Federal de Ciências da Saúde de Porto Alegre, Porto Alegre 90050-170, Brasil; 15 Hospital Albert Einstein, São Paulo 05652-900, Brasil; 16 Hospital de Base da Faculdade de Medicina de São José do Rio Preto, São Paulo 15090-000, Brasil; 17 Santa Casa de São José do Rio Preto, São José do Rio Preto 15025-500, Brasil; 18 Hospital Erasto Gaertner, Pontifícia Universidade Católica do Paraná, Curitiba 81520-060, Brasil; 19 Universidade Federal do Rio Grande do Norte, Natal 59300-000, Brasil; 20 Instituto do Coração, Universidade de São Paulo, São Paulo 05403-900, Brasil; 21 Medical Oncology, AC Camargo Cancer Centre, São Paulo 01509-010, Brasil; 22 Disciplina de Gastroenterologia, Universidade Federal de São Paulo, São Paulo 04021-001, Brasil; 23 Hospital São Rafael, Salvador 41253-190, Brasil; 24 Departamento de Cirurgia do Aparelho Digestivo Alto e Hepato-Bilio-Pancreática, Hospital de Câncer de Barretos, São Paulo 14784-400, Brasil; 25 Departamento de Patologia da Faculdade de Medicina da Universidade de São Paulo, São Paulo 01246-903, Brasil; 26 Clinica AMO, Salvador 1950-640, Brasil; 27 Hospital São José, São Paulo 01323-001, Brasil; 28 Universidade Nove de Julho, São Paulo 02111-030, Brasil

**Keywords:** neuroendcrine tumours, cancer, chemotherapy, targeted therapy, radionuclide peptide therapy, guideline

## Abstract

Neuroendocrine tumours are a heterogeneous group of diseases with a significant variety of diagnostic tests and treatment modalities. Guidelines were developed by North American and European groups to recommend their best management. However, local particularities and relativisms found worldwide led us to create Brazilian guidelines. Our consensus considered the best feasible strategies in an environment involving more limited resources. We believe that our recommendations may be extended to other countries with similar economic standards.

## Introduction and methodology

The World Health Organization (WHO) categorises neuroendocrine tumours (NETs) according to the tumour prognosis, considering the histopathology and proliferative index of tumours [1]. However, NETs are heterogeneous and may present with different clinical forms and behaviours. In this context, international guidelines have proposed treatment algorithms to facilitate better clinical management. Regional particularities should also be considered, which inspired the development of a Brazilian consensus. This paper presents the findings and recommendations of a panel of Brazilian specialists in various medical fields on the diagnosis, staging and treatment of NETs. All topics for discussion were previously selected and distributed among the members. The presentations and polls of experts on the panel took place during face-to-face meetings. The medical literature related to the selected subjects was assessed using MEDLINE (National Library of Medicine). Scientific evidence and recommendations were classified according to the CDC classification system (Tables 1 and 2). In the absence of sufficient evidence to obtain a clear conclusion, the final recommendations were made by consensus and voting, based on the opinion of the panel experts.

## Diagnosis and staging

### Pathology

NETs are tumours featuring neuroendocrine differentiation and may develop in various organs. The morphological classification proposed by WHO suggested three distinct prognostic groups: (1) grade 1 (G1) [Ki67 ≤ 2% and <2 mitoses/10 high-power fields (HPF)], (2) grade 2 (G2) (Ki67 3%–20% and 2–20 mitoses/10 HPF) and (3) grade 3 (G3) (Ki67 > 20% and >20 mitoses/10 HPF) [1]. G1 and G2 tumours are characterised as highly differentiated patterns with nested groups or pseudo-glandular arrangements. The poorly differentiated G3, which corresponds to the neuroendocrine carcinomas (NECs), may have a pattern of small or large cells.

#### Recommendations

- The pathology report should be used as the gold diagnostic standard.
- Immunohistochemistry (IHC) should be conducted for all suspected cases of NETs with a minimal panel of markers (low-weight cytokeratin, synaptophysin and chromogranin A) to confirm the neuroendocrine nature of the tumour [IVA], and reduce the chance of diagnostic errors.
- The proliferative index should be evaluated by counting mitotic figures using optical microscopy and the percentage of positive cells identified by IHC to determine the Ki67. The degree of determination requires a count of mitotically active cells in several areas, with at least 50 HPF (1 HPF = 2 mm^2^). The average of mitosis should be based on a count using 10 HPF. We recommend that the determined Ki67 should be expressed as a percentage of 500–2000 neoplastic cells counted in the areas of greater nuclear staining, known as hot spots. In a situation where a pathological analysis is performed using aspirated biopsy or a fine needle in which the cell count is below 500 cells, histologic grading is not recommended. Thus, the pathology report should be limited to confirming the neuroendocrine nature of the tumour.
- The proliferative index of Ki67 and/or mitotic cells can be done manually or with the aid of electronic programs for image analysis. The ratio of the percentage of positive cells over the total number of cells should be expressed in absolute numbers in the ICH report [2] [IVA].
- In the event of inconsistencies between mitotic count and Ki67, the higher histological grade should be used [1, 3] [IVA].
- Histologic degree of differentiation should be used to distinguish NETs as well differentiated (WD) or poorly differentiated, according to the architectural features (standard acinar, trabecular, solid, nests or diffuse) and cytomorphological (absence of pleomorphisms, small cells or large cells). We recommend that reporting the histologic degree of differentiation regardless of the proliferative index, i.e. NETs with a high proliferative index (>20%) should be described according to their cell differentiation and not solely as carcinomas [1, 3] [IVA].
- Poorly differentiated tumours should be classified in small or large cells [VB].
- Although there are many different staging systems for NETs, for example, the modified ENETS staging system for pancreatic NETs [4], for resected tumours, we recommend performing the pathological staging according to the guidelines in the American Joint Committee on Cancer, Seventh Edition [VA].
- Routine testing for other markers is not necessary [VE], except in cases of primary site determination where pancreatic markers (insulin, glucagon, etc) can guide the diagnosis of occult primary NETs [VB].
- Histological parameters should be described in all cases of primary tumour resection (i.e. surgical specimens, mucosectomias or excisions) [1–3, 5] [IVA].

#### Pathological reports should include

- Anatomical site;
- Diagnosis;Three-dimensional measurements of the lesion; Descriptions of unusual histology (oncocytic, clear cells, glands, etc);
- IHC results for neuroendocrine markers;
- Descriptions of multicentric disease;
- Descriptions of methods and systems used for grading;
- Mitotic index at 10 HPA or 2 mm, and counts at 50 HPA for hot spots;
- Descriptions of Ki67 indices in percentage values. A visual counting method is appropriate, but counts using printed images and/or by using software are preferable. Ki67 index, counting multiple hot spots for biopsies where the diagnosis of NECs cannot be excluded;
- Descriptions of vascular and perineural invasion;
- Descriptions of metastastatic lymph nodes, given as a ratio (number of affected nodes / number of examined nodes);
- TNM staging (refer to guidelines);
- Surgical margins, given as the distance between the tumour and the margin, if the distance is <0.5 cm;
- Proliferative changes or other abnormalities;
- Distance of invasion
  - Stomach: Depth of invasion in the gastric wall;
  - Small intestine: Depth of the intestinal wall invasion;
  - Colon: Depth of invasion in the wall of the large intestine;
  - Appendix: Depth of invasion in the wall of the appendix; description of presence of invasion extended to mesoappendix;
  - Pancreas: Presence of extra-pancreatic extension or bile duct invasion, duodenum or ampoule;
  - All sites: Involvement of serous or peritoneal surface, invasion of structures and/or adjacent organs.
- Presence of other non-neuroendocrine components;
- Presence of non-ischemic tumour necrosis.

#### Resection or biopsy of metastatic tumours

- Location and number of metastases;
- Extension of the involvement of the resected tissue, as a percentage;
- Dimensions of the largest metastasis;
- ﻿Presence of unusual histology (oncocytic, clear cell, glands, etc);
- IHC of neuroendocrine markers;
- Identification of the primary site by IHC;
- Grading;
- Mitotic index at 10 HPF or 2 mm; 50 HPF count;
- Ki67 index;
- Presence of other non-neuroendocrine components;
- Presence of non-ischemic tumour necrosis;
- For resected cases, describe the surgical margin as the distance between the tumour and the margin, if the distance is <0.5 cm.

### Utility of hormones, peptides, chromogranin A, 5-HIAA, gastrin and insulin in the diagnosis of NETs

#### Chromogranin A

Chromogranin A is acceptable, despite its limitations, as a general marker of NETs. The sensitivity and specificity limitations of chromogranin A may depend on the test used and clinical situations, such as secondary hypergastrinemia, gastrinoma, atrophic gastritis, *Helicobacter pylori* infection, use of proton pump inhibitors and liver or kidney dysfunction. Elevated levels of chromogranin A may also be caused by other neoplasias, diminishing its specificity [6] (Table 3).

#### Recommendations

- Chromogranin A can be used as a prognostic test [IIIC], and can be used for monitoring purposes [7] [IVC]. It should not be used alone to guide management.
- Repeated chromogranin A measurements can be made to assess tumour response [8], although the results should not be used to determine management [IIIC].

### 5-hydroxindoleacetic acid

**5-hydroxindoleacetic acid** (5-HIAA) is a metabolite of serotonin associated with carcinoid syndrome, being useful for initial and follow-up evaluations. However, high levels of 5-HIAA are also observed in patients with celiac disease, intestinal stasis, cystic fibrosis and chronic use of drugs that enhance serum serotonin (serotonin reuptake inhibitors and tramadol), while low levels are observed in patients with impaired renal function (Table 3).

#### Recommendations

- Patients should receive a low-serotonin diet for 48 h prior to and during collection of 5-HIAA in the urine (24 h) to avoid overestimation, as well as the withdrawal of serotoninergic drugs (e.g. antidepressants), if possible [VA].
- The 5-HIAA in the urine (24 h) should be measured in patients with carcinoid syndrome [IIA] and in all patients with metastatic midgut NETs involving the liver, even in the absence of specific symptoms [IVB].
- Dosage is optional for prognostic purposes [9] [IIIC].
- For patients receiving somatostatin analogues (SAs), 5-HIAA should be measured immediately before the next dose, in order to avoid artificially low values. [VA]

### Insulin

#### All recommendations are IVA

Measuring serum insulin, proinsulin and C-peptide following prolonged fasting (72 h) during hypoglycemia is considered the gold standard for diagnosis of insulinoma. However, the fasting test has limited application, because it requires hospitalisation to monitor blood glucose. In practice, documentation of clinical and laboratory hypoglycemia in the presence of Whipple’s triad accompanied by hypersinsulinemia is usually sufficient and recommended for diagnosing insulinoma.

The absolute values of glucose and insulin are the most relevant data, and any measurable insulin ≥ 3 mcU/ml ≥ 20, 8 pmol/l when blood glucose levels fall to values ≤ 55mg/dl or 3 mmol/l should be considerable abnormal [10, 11]. The final proinsulin dose in the fasting test shows high sensitivity and specificity for the diagnosis of insulinoma when concentrations are higher than 22 pmol/l. Patients with insulinoma show significantly lower, non-overlapping proinsulin levels compared with individuals without it [12, 13].

### Gastrin

#### All recommendations are IVA

Gastrin levels are useful for differentiating various types of stomach NETs [14]. Type I: Good prognosis associated with atrophic gastritis, usually multiple, presence of hypergastrinemia, and vitamin B12 deficiency; Type II: uncertain malignant potential, usually multiple, associated with hypergastrinemia in patients with gastrinomas and/or multiple endocrine neoplasias (MEN-1); Type III: Aggressive, often isolated and gastrin normal levels. It is also important to determine gastrin levels in cases of suspected gastrinoma (Table 3). Provocative tests or stimulation with calcium or secretin may be indicated when the gastrin concentrations are between 200 and 1000 pg/ml [15]. Other tests for specific hormones secreted by NETs should follow clinical indications.

#### General Recommendations for Functioning NET

- We do not recommend hormonal screenings for all patients in the absence of clinical indications, except for urinary 5-HIAA in asymptomatic patients with liver metastatic midgut NETs [VA]. In these cases, the identification of subclinical elevations of this marker may be useful for monitoring carcinoid heart disease (CHD).
- In rare cases of atypical carcinoid syndrome and 5-HIAA concentrations are in the upper normal limits, platelet serotonin measurement may be useful [IVC].

### Conventional imaging tests

The most commonly used conventional imaging tests for NETs are ultrasound (US), computed tomography (CT) and magnetic resonance imaging (MRI). US tests have extensive variability in the detection of pancreatic NETs (17%–79%), making this method unsuitable for identifying NETs [16]. However, endoscopic US (EUS) has shown an approximately 90% of detection rate for NETs in the head or pancreatic neck and duodenum [17]. CT studies with biphasic protocol showed 94% of sensitivity, and can detect pancreatic tumours at a rate similar to EUS. The combined sensitivity of the two methods has been described as 100% [18].

CT with multislice detector (CTMD) has an average detection capability of 73% (39%–94%) for pancreatic NETs [16]. False negative results can occur with tumours <2 cm and insulinomas. Thus, sequential evaluation with CTMD and EUS is recommended for pancreatic NETs, except in cases of suspected insulinomas [19]. CTMD with three-phase protocol is a useful tool for diagnosis and staging [20]. The EUS, ^68^Ga positron emission tomography (PET/CT^68^Ga) and CTMD have comparable accuracies in the diagnosis of duodenopancreatic NETs [21]. MRI has been found to be comparable with EUS in detecting duodenopancreatic NETs, and is recommended as the best method to use, employing the T2 and post-contrast T1 sequences in the arterial phase [22].

#### Recommendations

- US for primary assessment of NETs should not be used [16] [IVD].
- MRI, CTMD and EUS have a similar sensitivity to detect pancreatic NET [17, 18]. However, CTMD is preferred for surgical planning for better vascular assessment [20] [IVB].
- Three-phase CT is the preferred method for staging liver metastases from NETs [VA]. MRI can be used to evaluate liver metastases, if available [23] [IVB].
- CT enterography is optional and experimental [24] [IVC].

### Endoscopic ultrasound for diagnosing pancreatic and duodenal lesions

The sensitivity of EUS for diagnosing duodenopancreatic NETs is 87%, and its specificity is 98% [25]. For insulinomas, the sensitivity is 87.5% and the specificity is 97.4%. [25]. For gastrinomas, the sensitivity is 84.5% and the specificity is 95% [25]. In addition to diagnostic use, EUS can also be used with fine needle aspiration for histological studies [26].

#### Recommendation

- EUS can be used for the diagnosis of pancreatic or duodenal NETs [25] [IIA].

### PET/CT^18^F/FDG PET/CT^68^Ga-somatostatin analogue

The ^18^F-Fluorodesoxiglucose (^18^F/FDG) is a radiopharmaceutical analogue of glucose and PET/CT^68^Ga is an analogue (agonist) of somatostatin receptor type 2. While the performance of scintigraphy octreoscan is similar to multidetector CT [27], the accuracy of PET/CT^68^Ga is superior when compared with conventional imaging tests for WD NETs [28, 29]. The PET/CT^18^F/FDG has limited accuracy in staging WD NETs [30].

#### Recommendations for the use of PET/CT18F/FDG PET/CT68Ga-somatostatin analogue

- PET/CT^18^F/FDG can be used to stage for metastases of initially resectable G3 [31] [IVC], but should not be used to determine treatment.
- PET/CT^68^Ga-SA, if available, is indicated and preferable to octreoscan for staging G1 and G2 NETs [28, 29] [IIA].
- PET/CT^68^Ga-SA or octreoscan can be used to determine the somatostatin receptor expression. This is necessary for the indication of radiopharmaceutical therapy (see recommendations for radionuclide peptide therapy) [IIA].
- PET/CT^68^Ga-SA is recommended in cases of unknown primary lesions or suspicious lesions [32] [IIIA].
- PET/CT^68^Ga-SA can be used to evaluate suspected recurrences after surgery [33] [IIIA].

### Whole-body magnetic resonance

Whole-body magnetic resonance (WBMR) employs three sequences: (1) STIR, (2) T1 (3) and diffusion. Its advantages include lower scanning costs and absence of ionising radiation or intravenous contrast administration. PET/CT^68^Ga apparently has superior sensitivity compared with WBMR for detecting WD NETs, specifically in bone and primary lesions [29].

#### Recommendations

- WBMR can be used to stage WD NETs when PET/CT^68^Ga is not available [29] [IIIC].
- WBMR, if available, is a method with high accuracy for screening patients with NETs without ionising radiation [34] [IIIB].

### Extended examinations of unknown primary tumours

Patients with unknown primary tumours are characterised as such when conventional tests such as CT, MRI, functioning peptide dosage, endoscopy and colonoscopy cannot determine NET origin. The importance of identifying an occult primary NET relies on the potential to impact patient survival [35–37], for example, by indicating the need for a liver transplant.

Among the methods of examination, surgery itself is an option where the primary tumour can be identified intraoperatively [38]. Enteroscopy has low accuracy in detecting NETs of the small intestine (sensitivity of 33%) [39]. As for functional tests, PET/CT^68^Ga is considered an excellent imaging method for detecting occult primary tumours [40–43]. An observational study showed that PET/CT^68^Ga identified 59% of occult NETs [41]. If PET/CT^68^Ga is not available, the SRS (octreoscan or 99mTc-octreotide SPECT/CT) can be used [44], albeit with less accuracy.

﻿Enterotomography can be considered in selected cases [45]. The use of endoscopic capsule may also be considered, although its sensitivity varies widely in the literature (45%–80%) for detecting NETs [46, 47].

#### Recommendations

- EUS can be used in cases of suspected primary tumours in the stomach, duodenum and pancreas.
- PET/CT^18^F/FDG can be used in cases of suspected high-grade primary tumours [VD].
- Surgery can detect primary tumours intraoperatively independently of preoperative location [VC].
- PET/CT^68^Ga is one of the most accurate methods for detecting WD occult NETs [32, 40–43] [IIIA].
- If PET/CT^68^Ga is not available, we suggest using the SRS (octreoscan or 99mTc-octreotide SPECT/CT) [IVB].
- Enterotomography can be considered, particularly in cases of suspected Crohn’s disease [45] [VC].
- Endoscopic capsule [47] [IVC] and enteroscopy [VC] can be considered for occult small bowel NET examinations. Be aware that endoscopic capsule may cause bowel obstruction up to 2% of cases [48].
- IHC in the investigation of occult NETs can be useful, especially in occult pancreatic NETs (see pathology section) [VC].

### Patient follow-up

There are very few studies to guide the follow-up of patients with NETs. Most recommendations have been based on expert opinions [49].

#### Recommendations

- NET G3: Clinical assessment without radiological tests should be performed every three months for two years and every six months from the third to fifth year [VB]. Conventional imaging (CT and MRI) of the abdomen, pelvis and chest can be used in cases of suspected recurrence.
- Gastrointestinal and pancreatic NET G1 or G2 treated with R0/R1 resection: Clinical and imaging tests (CT and/or MRI) should be performed every 4–6 months for two years. After this period, perform annual examinations every 3–5 years or earlier if there is suspicion of relapse [VB].
- Tumours <1 cm require monitoring only in the presence of negative factors of prognosis, such as poorly differentiated histology or high mitotic index [IIIB].
- Functional tests such as octreoscan and PET/CT^68^Ga are not recommended for follow-up in patients with no evidence of disease as determined by conventional imaging [VE].
- Chromogranin A measurement alone is not recommended [IVE].

### Follow-up for patients with evidence of metastatic disease receiving treatment or watchful wait

Because there is no level 1 evidence for the follow-up of patients with NETs, there is room for personalisation of frequency of follow-up visits and imaging tests.

- Conventional imaging tests (CT and/or MRI) should be performed every 3–6 months [VA].
- In cases of rapid disease progression or dubious examination results, a new liver biopsy may be considered to reassess proliferation [VC].

### Follow-up for patients with specific NETs

#### Gastric

- Type 1: Endoscopic assessment every 12 months in recurrent patients and every 24 months in non-recurrent patients [VB]. There was no consensus on the follow-up period. The follow-up should be based on clinical judgments in individual cases.
- Type 2: Consider the same recommendations for multiple endocrine neoplasia (see MEN section).
- Type 3: CT thorax, pelvis and abdomen should be performed every 3–4 months for the first two years and every 6–12 months from the third to fifth year [VB].

#### Colon and rectum

- Colorectal NETs <1 cm G3 and G1–G3 from 1 to 2 cm: Annual monitoring according to the protocol for adenomatous polyps [50].
- For cases of NETs >2 cm, we recommend annual colonoscopies for five years.
- Octreoscan or PET/CT^68^Ga should not be included in the routine follow-up.

## Surgical treatment

### Resection of the primary tumour: resectable disease

#### Recommendations

- We recommend complete resection with curative intent for all NETs.
- ForWD pancreatic NETs <2 cm, particularly G1 tumours, we recommend surveillance, with CT scan performed every six months [IVB]. For growing tumours and/or those with higher ki67 index, surgery is recommended [51–53] [IVB].
- Midgut NETs should be treated with resection of mesenteric lymph nodes in order to prevent mesenteric fibrosis [VB].

### Resection of the primary tumour in metastatic disease

The practice of resection of the primary NET in the metastatic setting is based on small retrospective series. In general, patients with good performance status and symptomatic primaries have indication for resection. Patients with liver transplant indications should also have the primary tumour resected. In asymptomatic metastatic patients, surgery for primary G1 or G2 tumours of the small intestine should be considered for the risk of obstruction, mesenteric fibrosis and intestinal ischemia [54].

#### Recommendations

- Resection of G1 and G2 midgut primary symptomatic NETs, with or without involvement of mesenteric and symptomatic primary colon or rectum NETs [55] [IVA].
- Resection of the pancreatic or gastric NET primary in the metastatic disease setting is not recommended [56] [IVE].
- Resection of asymptomatic primary tumours of the midgut can be considered in select cases, where the tumour presents a significant risk for complications such as obstruction and mesenteric fibrosis [54] [IVC].

### Surgical treatment of liver metastasis: recommendations for resectable disease

Patients with G1 and G2 NETs presenting with metastases limited to the liver may be considered for resection of entire tumour. In order to obtain R0 resection, combined radiofrequency ablation should be considered for local control [57]. In general, resectable liver lesions are those that can be extracted with free margins and are maintaining at least two contiguous liver segments and their vascular pedicles, and biliary and venous drainage. In these cases, parenchymal preservation techniques such as portal embolisation [58] and staged hepatectomy [58] should be discussed with an experienced surgical team. While retrospective series have reported encouraging survival rates for NET patients undergoing hepatectomy [59, 60], overestimation for selection bias should be considered.

#### Recommendations

- The decision for liver resection of metastatic WD NETs should be discussed by multidisciplinary teams [VA]. R0 resection should be aimed whenever possible [57] [IVB].
- Ablative techniques such as radiofrequency can be combined with surgery in order to obtain negative margins. Intraoperative US [IVA] should be performed for staging liver lesions [57].
- Liver resection should be performed by experienced teams [VA].
- A simultaneous approach to primary and liver tumours can be considered [61] [IVB].

### Surgical treatment of liver metastasis: recommendations for unresectable liver predominant disease

#### Debulking surgery

Debulking is indicated for functioning NETs to alleviate symptoms not adequately controlled with systemic therapy and/or locoregional therapies of the liver [62–64]. The magnitude of debulking surgery is arbitrary but considered by most as at least debulking >90% of liver lesions. The debulking of extrahepatic metastatic disease is not recommended. If the patient has CHD, heart surgery must be performed before surgical debulking for the high risk of intraoperative bleeding.

#### Recommendation for surgical debulking

- Recommended for patients with good performance status, functioning WD NETs, with predominant liver disease and poorly controlled clinical syndrome despite systemic and/or locoregional therapies [62, 64] [IVB].

## Locoregional treatment

### Embolisation and chemoembolisation

Patients with WD NETs limited to the liver or predominantly unresectable hepatic disease can benefit from locoregional therapies, including liver embolisation, combined or not with chemotherapy and percutaneous radioablation (with radioisotopes, cryoablation or radiofrequency). Contraindications for hepatic embolisation are prior pancreatic and/or biliary surgery, portal vein thrombosis and those with moderate-to-severe liver dysfunction.

Retrospective studies have evaluated the benefits of embolisation with reported symptomatic responses in 80% of cases and objective responses in approximately 30% [65]. Although there are no controlled studies comparing embolisation with chemoembolisation, we prefer chemoembolisation for pancreatic NETs, given their higher sensitivity to chemotherapy in comparison with midgut tumours. The most frequently used drugs in chemoembolisation are doxorubicin, mitomycin and cisplatin. Common symptoms of post-embolisation and post-chemoembolisation syndromes include nausea, vomiting, increased liver enzymes, abdominal pain and fever, and myelosuppression if combined with chemotherapy. Fever may persist for up to 30 days depending on the extension of tumour necrosis, also characterised as an inflammatory response to trauma. It is important to consider that patients with moderate hepatic impairment, previous reconstruction of the biliary tract or portal vein obstruction are contraindicated for liver embolisation, with or without chemotherapy, for the high risk of liver impairment and infections.

#### Recommendations

- Liver embolisation and chemoembolisation are safe and are indicated in symptomatic inoperable metastatic liver disease [65] [IIB] or asymptomatic, but progressive, disease [66] [IIIC].
- We prefer pure embolisation for midgut G1 NETs, limiting the addition of chemotherapy to cases of G2 pancreatic or G2 gastrointestinal NETs [VB].
- Both embolisation and chemoembolisation may be repeated, if no significant liver dysfunction is detected [VB].

### Radiofrequency ablation

Radiofrequency ablation is associated with symptomatic and/or radiological response in 70%–80% of NET patients, with an overall five-year survival rate of 50% [67]. Radioblation may be performed by a percutaneous approach and can be considered for patients with inoperable diseases limited to the liver, with lesions <3–4 cm. Radioablation may also be considered intra-operatively, with the goal of achieving R0 surgery.

#### Recommendations

- Percutaneous radiofrequency ablation can be considered for palliation of WD inoperable and progressive NET patients with a low volume of liver metastasis [67] [IVB]. The best results can be achieved in patients with up to 5 nodules (<3 cm).
- Ablation with open radiofrequency can be used as a complement to surgery, in an attempt to achieve free margins and/or manage lesions with difficult resectability [57] [IVC].

### Radioembolisation

Radioembolisation is a new transcatheter therapy that uses ^90^Yttrium as the radiation-emitting radioisotope. ^90^Yttrium releases beta radiation (β) with an average tissue penetration of 2.5 mm and a maximum penetration of 11 mm. Radioembolisation offers doses at 100–300 Gy, which would not be possible through external radiotherapy due to liver toxicity [68]. Post-embolisation syndrome affects 40%–50% of patients [65] and, in most cases, has low morbidity. Serious side effects, such as hepatitis, gastritis, peptic ulcers, pancreatitis, cholecystitis and pneumonitis, are rare, occurring in 2%–8% of cases. Hepatic disease induced by radiation is even more rare (<1%). In G1 and G2 NETs, retrospective studies show varying results for disease control [69, 70].

#### Recommendation

- Radioembolisation with ^90^Yttrium can be used as a rescue treatment for progressive liver disease, but should be used only in select cases of patients with G1 and G2 NETs, who have good hepatic reserve, and have been previously treated with locoregional and systemic therapies [69, 70] [IIIC].

### Unresectable disease limited to liver: liver transplant

Liver transplantation may be a potentially curative option for selected patients with WD NET. Eligible cases for transplantation, however, should be very well selected [71–74].

- Liver transplantation may be considered in select cases for patients with G1 and G2 NETs, preferably with Ki67 < 10%, at least six months of stable, good performance status, minimum comorbidities, no extrahepatic disease and preferably when other treatment options have been exhausted [71, 73] [IVC].
- Assessments to rule out extrahepatic diseases should include total abdomen CT/MRI, chest CT, octreoscan or preferably, if available, PET/CT^68^Ga [VA].
- Eligible patients must have the primary tumour resected prior to transplantation.

## Systemic treatment of gastroenteropancreatic NET

### Somatostatin analogues as an anti tumour therapy versus watchful waiting

Two phase III placebo-controlled studies have demonstrated the antitumour effects of SAs (octreotide and lanreotide) in WD gastroenteropancreatic (GEP) NETs with Ki67 < 10% [75, 76], regardless of NET functionality.

#### Recommendations

- SAs are the first-line systemic treatment of choice for WD, inoperable and advanced progressive WD GEP NETs, with Ki67 < 10% [75, 76] [IB].
- We favour the confirmation of SSTR2 expression through octreoscan or PET/CT^68^Ga prior to SA-based treatment [IIB], although this is not mandatory [77].
- We consider octreotide and lanreotide to have similar safety and efficacy for treating WD GEP NETs [VB].
- Watchful waiting can be recommended in WD NETs, preferably those with G1, non-functioning, low-volume, and oligosymptomatic disease [VC]. WD G1 NETs are indolent and may stabilise for months to years without treatment as observed in the median of progression-free survival (PFS) in the placebo arm of the CLARINET study [75]. Furthermore, there is no evidence that SAs either have an impact on overall survival (OS) or on quality of life in patients with non-functioning tumours.

### Interferon-α

Despite its unfavourable toxicity profile, interferon-α is still used and, in many low-resource countries, it is one of the few available treatments for NET. Several studies have shown that interferon-α provides tumour control, mostly through disease stabilisation and symptomatic response in carcinoid syndrome [53]. However, randomised studies have not shown that interferon-α alone or combined with SA was superior to monotherapy [53].

#### Recommendation

- Interferon-α can be used in cases of SA-resistant and progressive WD NETs [53, 78] [IC].

### Chemotherapy for WD NETs

Chemotherapy, although not studied in phase III trials, is considered an effective treatment for G1 and G2 pancreatic NETs, with the response rates of 30%–40% with temozolomide and capecitabine [79], capecitabine and oxaliplatin [80], FOLFOX, dacarbazine or streptozotocin alone or associated with 5FU. The best regimen or the number of cycles has not been established, and this decision should consider limiting toxicities and maximum response. Chemotherapy has a limited role in patients with WD metastatic midgut NETs [79]. Predictive factors for chemotherapy responses have been studied. Methyl-guanilmetiltransferase (MGMT) deficiency as assessed by IHC or by methylation may have a predictive role for determining the alkylating response [81]. However, the best MGMT measurement method has not been determined. The role of Ki67 as a predictor of response to chemotherapy in WD NET is not established, but may be considered for chemotherapy indication.

#### Recommendations

- Patients with radiological and/or symptomatic progressive WD pancreatic NET [80] [IIB].
- Patients with WD gastrointestinal NET who have exhausted other treatment options [80] [IID].
  - Suggested regimens: Capecitabine combined with temozolomide or oxaliplatin, FOLFOX and streptozotocin with or without 5FU.
- Increased expression of Ki67 can be used to recommend chemotherapy in WD GEP [VB].
- MGMT expression should not guide the use of temozolomide/dacarbazine [81] [IVD].

### Targeted therapy in non-pancreatic gastrointestinal NET

#### Everolimus

Everolimus, an oral inhibitor of mTOR, was effective in WD pancreatic, gastrointestinal or pulmonary NETs. The RADIANT-2 study [82], a phase III, placebo-controlled, double-blind study of patients with WD functioning gastrointestinal and pulmonary NETs, found that 10 mg of everolimus continuously combined with 30 mg of intramuscular octreotide LAR prolonged PFS (median: 11.3 months to 16.4 months; hazard ratio (HR): 0.77; IC 95%: 0.59–1; p = 0.026). This value was not statistically significant by radiology central review, but suggested benefit by local evaluation. The benefit in PFS in gastrointestinal and pulmonary NET patients treated by everolimus was formally demonstrated by the RADIANT-4, a phase III trial of similar design, which included only patients with non-functioning tumours [83].

#### Recommendations

- Everolimus monotherapy is recommended for non-functioning and progressive gastrointestinal or pulmonary WD NETs [IA]. Everolimus may be used as a first-line or second-line treatment after SA [83] [IIB].
- Everolimus in combination with SA is recommended for patients with functioning tumours [IIA]. Utilisation is preferable after progression on SA monotherapy [82].
- Everolimus can be used as a monotherapy in functioning tumours to control hormonal syndromes [78, 84] [IVC].

### Sunitinib

Sunitinib is an oral inhibitor of tyrosine kinase receptors, including PDGFR, VEGFR and c-KIT. A phase II study showed limited efficacy for sunitinib in treating gastrointestinal NETs, with the median PFS rate of 7 months in carcinoid tumours [85].

#### Recommendation

- Sunitinib is not recommended for patients with gastrointestinal non-pancreatic NETs [85] [IID].

### Targeted therapy in WD pancreatic NET

The phase III placebo-controlled RADIANT-3 trial for patients with advanced, progressive pancreatic NET showed significantly prolonged PFS in favour of everolimus, with a median of 11 months versus 4.6 months (HR: 0.35; IC 95%: 0.27–0.45; p < 0.0001) [86]. Similarly, a placebo-controlled phase III study of sunitinib for the same population WD demonstrated the median PFS rate of 11.4 months versus 5.5 months (HR = 0.42; IC 95% = 0.26–0.66; p < 0.001) [87].

In pancreatic NETs, the choice between the two drugs should consider their toxicity profiles. Serious adverse events (G3 and grade 4) most commonly associated with sunitinib were neutropenia (12%), hypertension (10%), and hand and foot syndrome (6%), while everolimus commonly promoted stomatitis (7%), anemia (6%) and hyperglycemia (5%) [83, 87]. In a meta-analysis of individual data, severe pneumonitis related to everolimus occurred in approximately 2% of patients [88]

#### Recommendations

- Everolimus and sunitinib monotherapy are recommended for WD functional or non-functional, progressive advanced NETs [86, 87] [IA].
- Both drugs can be used in first-line therapy or following SA and/or chemotherapy [86, 87] [IIIA].
- Everolimus and sunitinib can also be combined with SA to treat functional tumours [86, 87] [IIA].
- In metastatic insulinomas, the use of everolimus is preferred drug [IVA].

### Radioisotopes

Radioisotopes, also termed peptide radionuclide receptor therapy (PRRT), have been successfully used to treat patients with metastatic WD NETs, with somatostatin receptor positive expression confirmed by scintigraphy with ^111^Indium-labeled octreotide or PET/CT^68^Ga [89]. Contraindications for PRRT include pregnancy, severe psychiatric disorders, moderate or severe renal impairment and low bone marrow reserve.

In a large retrospective series of 500 patients with pancreatic or gastrointestinal NET treated with ^177^Lutetium, the overall response rate was 18% in the intention to treat population, with the median PFS rate of 33 months [90]. Patients were treated with maximum cumulative dose of 800 mCi. Treatment was well-tolerated with acute toxicity within 24 h of application that included nausea, vomiting and abdominal pain. Delayed toxicities may occur, particularly with PRRT with ^90^Itrium, with the most common events including hematologic (secondary myelodysplasia or leukemia), kidney failure and liver toxicity [89].

A recent study (NETTER-1) randomised 229 patients with G1 or G2 progressive midgut NET after SA to receive 60-mg octreotide LAR or ^177^Lutetium associated with 30-mg octreotide LAR [91]. Outcomes favoured PRRT, with the response of 19% versus 3%, median PFS not reached (estimated at 40 months) versus 8.4 months (HR: 0.2; p < 0.0001) and preliminary data suggests increased OS.

#### Recommendations

- ^177^Lutetium is recommended for progressive WD midgut NET [79] [IA].
- The best timing to administer ^177^Lutetium has not been established, although improved PFS has been demonstrated in the second-line setting. For limited data on delayed safety analysis of this treatment and perceptions of greater toxicity of chemotherapy and targeted-therapy after ^177^Lutetium, we prefer to indicate it after other systemic therapies have failed [VB].
- ^177^Lutetium is recommended for patients with an advanced WD pancreatic or hindgut NET whose disease progressed with SA, locoregional and/or systemic therapy [90] [IIIB].
- It is necessary to confirm SSTR2 expression by octreoscan or PET/CT^68^Ga prior to PRRT [79, 89, 90] [IA].

### Management of bone metastases

Bone metastases occur in 18%–46% [92, 93] of WD NET and are associated with worse prognosis [94]. For management, we recommend specific treatments of NETs plus bisphosphonate or denosumab [95] at the physician’s discretion.

#### Recommendations

- There is no preferred imaging method for the diagnosis of bone metastases. Although PET/CT^68^Ga is the most accurate imaging method to stage bone metastases in WD NETs [96, 97], different imaging techniques can be used, such as bone scintigraphy and/or octreoscan, or whole body diffusion MRI [IVB].
- Bisphosphonates or RANK ligand inhibitors, such as denosumab, can be used in patients at risk for complications from bone metastasis [95] [IVC].

## Management of hormonal syndromes and its complications

### De novo and refractory carcinoid syndrome

Control of the carcinoid syndrome is important not only for the improvement of symptoms and consequently the patient’s quality of life, but also to prevent or delay the development of complications associated with carcinoid syndrome, such as retroperitoneal fibrosis and carcinoid cardiopathy [98]. SAs have been the first-line standard treatment of carcinoid syndrome for decades. However, symptomatic progression occurs, and various therapies have been tested in small phase II studies or retrospective series to treat refractory carcinoid syndrome [78]: (1) dose-escalation of SA, (2) interferon-α, (3) everolimus, (4) radioisotopes, (5) locoregional therapies and (6) liver debulking surgery. Recently, an oral inhibitor of the serotonin synthesis, telotristat etiprate, has shown better control of number of bowel movements per day in a phase III placebo-controlled trial [99]. The drug is not yet approved, and although current formal recommendations are difficult to make, it is likely that telotristat will be placed early in the treatment sequence of refractory carcinoid syndrome [78]. Chemotherapy has limited effects in midgut tumours, which are the most common type associated with carcinoid syndrome.

#### Recommendation for the treatment of carcinoid syndrome *de novo*

- We recommend the use of SA [IA]. We consider octreotide and lanreotide interchangeable for controlling carcinoid symptoms or other symptoms associated with hormonal syndromes in NET.

#### Recommendations for the management of carcinoid syndrome uncontrolled with SA label dosages.

- Physical examination should be performed to discard absorption problems (e.g. fibrosis in application site) [VA].
- Increased doses of SA can be used [78] [IIB]. There was no consensus on the recommended strength. There is little evidence to recommend the use of higher doses (>40 mg) of octreotide LAR. There is no evidence to support the use of increased doses of lanreotide (120 mg or more).
- Shorter intervals between injections for patients whose symptoms recur before next injection [VA]
- Everolimus [IVC] or associated with SA [78] [IIC].
- Interferon-α combined with SA [78] [IIIC].
- ^177^Lutetium or ^90^Itrium radioisotopes can be indicated for NETs with proven expression of SSTR2 [IVB] or I^123^-metaiodobenzylguanine (MIBG) in NET with proven expression of MIBG [78] [IVB].
- Surgical debulking of the liver [78] [IVB].
- Locoregional therapies such as embolisation of hepatic artery or radiofrequency ablation in liver predominant disease [78] [IIIB].

### Glycemic control in insulinoma

Hypoglycemia caused by insulinoma can be fatal. Therefore, glucose control is extremely important, both in the preoperative period (when the vast majority of insulinoma are benign) and unresectable, or metastatic setting. Patients with insulinoma should be hospitalised for management of hypoglycemia.

#### Recommendations

- Hospitalisation for control of glycaemia with frequent meals at least every 3 h, continuous infusion of dextrose and glucose monitoring.
- Diazoxide for glycemic control [VB] can be used. However, this drug is not widely available.
- SA can be used cautiously for control of glycemia [VB]. This approach should be done carefully as regulatory hormones may be blocked leading to severe hypoglycemia. SA is recommended when SST2 expression is confirmed. Treatment should be initiated with short-acting octreotide for few days to better assess glycemic control. If effective, long-acting SA may be used.
- Everolimus has antitumour and hyperglycemic effects in metastatic insulinomas [84] [IVA].
- Verapamil and glucocorticoids are optional in selected refractory cases [VC].
- Treatment with targeted therapy or chemotherapy should be performed in combination with glycemic support [VA].

### Management of carcinoid heart disease

CHD is among the most dramatic complications from carcinoid syndrome, as it is associated with poor prognosis [98]. The peptides released into the blood, particularly serotonin and other tachinines, mainly affect the right-hand side of the heart, damaging the tricuspid valve in 97% of cases [98]. Echocardiography with Doppler is the recommended diagnostic exam, allowing for the observation of mild regurgitation of the mitral valve, dilation of the right chambers and tricuspid valve insufficiency.

#### Recommendations

- We recommend screening with echocardiography in patients with elevated 24-h urine 5-HIAA, regardless of carcinoid symptoms [VA].
- The echocardiogram should be performed by a professional familiar with CHD [98] [IIIA], and this should include evaluation of patency of *forame ovale* by the bubble test. The measurement of NT pro-BNP, a predictor of right-hand-side heart overload is optional [98] [IIIC].
- In patients without CHD, an echocardiogram should be performed annually or when there at onset of symptoms of cardiac congestive failure [VA].
- Detailed assessment of the CHD severity can be performed by other diagnostic modalities (MRI, etc) [100] [IVC].
- Patients with CHD should be managed by a multidisciplinary team of oncologists, cardiologists, endocrinologists and surgeons [VA].
- Pharmacological and non-pharmacological treatment of heart failure, such as water and salt restriction, diuretics and digitalic drugs, can improve symptoms but do not alter clinical outcomes [VB].
- The definitive treatment of CHD is surgical valve replacement [IIA]. Surgery should be considered if the patient experiences symptoms of heart failure, right ventricular dilatation and decline of right ventricular function [98, 101]. Patients with poor performance status, metastatic disease and/or difficult to control carcinoid syndrome should not be considered candidates for valve replacement [IID].
- Valve repair, compared with valve replacement, should be avoided because of the risk of post-repair stenosis and significant valve damage associated with underlying carcinoid syndrome [IVD].
- Valve replacement, when indicated, should be performed in centres with experience in the treatment of NETs [VB].
- The use of biological valves (bioprosthesis) is preferred for the lower risk of bleeding compared with metallic valves [98, 101] [IVB].
- Avoid the use of opioids, neuromuscular inhibitors, adrenaline, noradrenaline, dopamine and isoproterenol during anesthetic induction in patients with CHD.
- We recommend the use of intraoperative IV octreotide beginning at least two hours before surgery, with continuous infusion for 48 hours after surgery [IIIB] with the goal of preventing carcinoid development [98].

### Clinical management of glucagonomas, gastrinomas and vipomas

#### Glucagonoma

Glucagonoma is an aggressive pancreatic NET of glucagon-producing cells that often present with metastatic disease. Clinical manifestations include anemia, weight loss, diabetes and dermatological features of a typical necrolytic migratory erythema. They may also present stomatitis, glossitis, diarrhea, abdominal pain, psychiatric disorders and venous thromboembolism.

#### Recommendations

- Surgical treatment, if resectable, and the use of SA as a symptomatic treatment of clinical syndrome of glucagonoma.
- Consider parenteral nutrition, vitamin supplementation, assessments of the presence of depression and prophylactic anticoagulation in all patients with glucagonoma [VB].

### Gastrinoma

Gastrinomas are characterised by ectopic secretion of gastrin from pancreatic or duodenal NET, and resultant gastric ulcers. Severe peptic ulcers with gastroesophageal reflux and diarrhea are characteristic of Zollinger–Ellison syndrome.

#### Recommendations

- We recommend the use of high doses of proton pump inhibitors, with or without H2 receptor blockers [102] [IVA].
- SA [VA] as an antitumour therapy can be used to control associated diarrhea.

### Vipoma

Vipoma is a rare pancreatic NET that produces vasoactive intestinal peptide, an important peptide in the neuromodulation of intestinal function. Clinical manifestations include intense watery (choleric) diarrhea, with fluid and electrolytes depletion, risk of acidosis and hypovolemic shock, reductions gastric acid secretion, hyperglycaemia, hypercalcemia and flushing.

#### Recommendation

- We recommend SA as the first treatment [IVA]. It is important to manage blood volume, hypocalcemia and acidosis [103].

### Neuroendocrine carcinomas or G3

NECs or G3 are rare and associated with poor prognosis, with a median OS of less than a year [104]. Retrospective studies suggest OS gains with adjuvant therapy [105]. For metastatic disease, platinum-based chemotherapy is considered standard [104], without apparent clinically relevant differences between cisplatin and carboplatin [106]. Recently, retrospective series have shown that G3 tumours may present WD histologies and that this subgroup has a lower proliferative index and better prognosis compared with poorly differentiated carcinomas [107, 108].

A retrospective multicentre European study showed higher Ki67 index is associated with better response to platinum-based chemotherapy [106]. Ki67, however, was not found to be a predictor for response to chemotherapy in other studies [108].

### Localised G3

#### Recommendations

- Resectable G3 NEC should undergo oncological surgery [105] [IVA].
- Consider definitive treatment with platinum-based chemotherapy combined with radiotherapy for locally advanced or unresectable tumours [105] [IVA].
- Adjuvant chemotherapy with cisplatin (or carboplatin) associated with etoposide or irinotecan for 4–6 cycles can be considered in patients with good performance status after surgical resection [105] [IVC].

### Treatment of metastatic disease

#### Recommendations

- Cisplatin (or carboplatin) associated with irinotecan or etoposide for first-line treatment [104, 106] [IIIA].
- Cisplatin (or carboplatin) may be offered again to responders who have received their last dose more than three months before [VB].
- Temozolomide-based or oxaliplatin-based regimens may be used in WD G3 tumours [VC].
- Temozolomide or dacarbazine can be used as second-line treatments after platinum regimens [109] [IIIB].

## Common hereditary syndromes associated with GEP NETs: multiple endocrine neoplasia type 1 (MEN-1) and von Hippel–Lindau syndrome

GEP NETs may be associated with hereditary syndromes as MEN-1, von Hippel–Lindau (VHL) and neurofibromatosis-1, among others. In this consensus, we have focused on MEN-1 and VHL as they are the most commonly associated syndrome with NETs.

### MEN-1: clinical and molecular diagnosis

The inheritance pattern of MEN-1 is autosomal dominant with almost complete prevalence by 50 years of age. Patients with MEN-1 have high susceptibility to the development of tumours in the pituitary gland, the parathyroid glands, and duodenal endocrine cells or pancreatic islets [110]. The clinical diagnosis of MEN-1 involves tumours located in at least two of the three main sites (pituitary, parathyroid and pancreas/duodenum). The diagnosis of familial MEN-1 is defined beyond a clinical diagnostic index case of MEN-1 when a first degree relative presents a tumour diagnosis in at least one of the three main sites [111–113].

Genetic diagnosis is made in individuals with a *MEN1* germline mutation, whether or not they have any clinic manifestations related to MEN-1. However, it is essential that genetic counselling is carried out and that the DNA collection for mutation research on the *MEN1* gene is offered to all patients with the clinical diagnosis of MEN-1. Mutations not identified by Sanger sequencing analysis should be sought by deletion analysis by multiplex ligation-dependent probe amplification (MLPA) [113]. In specialised centres, it is possible to proceed with the investigation of other genes (*p15*, *p18*, *p21*, *p27* and *AIP*) in patients who are negative for the presence of mutation by the aforementioned techniques [113]. Unlike the fully proven benefit-risk genetic research value of the *MEN1* gene by Sanger sequencing and MLPA, the clinical value of analysing these other MEN-1-related genes has not yet been established, because they are exceptionally rare.

#### Considering that MEN-1 is a rare disease, the level of evidence for the recommendations is based on expert opinion [VA].

- Genetic analysis of the *MEN1*gene should be offered to symptomatic and asymptomatic first-grade relatives of the index case with a known mutation.
- Mutation detection in symptomatic cases and MEN-1-related tumours are important to exclude phenocopies (relatives who develop sporadic MEN-1-related tumours and, therefore, are negative for mutation found in the family). Phenocopies should receive genetic counselling, being informed of the absence of risk of disease transmission to descendants. In addition, the definition of phenocopies can result in change of surgical and clinical management, which follows the standard recommendation for sporadic cancers.
- We recommend clinical screening with hormonal and radiological tests for all patients with MEN-1 and asymptomatic family members recognised as having *MEN1* germline mutations. Early diagnosis allows for the best surgical treatment and potentially promotes increased survival and quality of life in patients with mutation.
- Clinical screening and laboratory tests of patients and asymptomatic carriers of *MEN1* mutation should be performed annually.
- Annual hormonal examinations for pituitary tumours should be initiated at 5 years old with prolactin measurements, growth hormones and IGF1, along with active clinical examinations for symptoms associated with insulinoma.
- The beginning of primary hyperparathyroidism (HPT) investigations should be initiated at 8 years old with total calcium dosage, ionised calcium and parathyroid hormone measurements.
- Active investigation of clinical symptoms of gastrinoma should be made beginning at 10 years of age. For asymptomatic cases, gastrin dosage should be done annually at 20 years old of age.
- Radiological investigations, including MRI of the pituitary, MRI of the abdomen and chest CT should be made from 5, 10 and 15 years old, respectively, for the investigation of pituitary non-functioning adenoma, non-functioning pancreatic tumours and bronchial/thymic tumours. The frequency of radiological images is not clearly defined, but we recommend that they be provided every 1–3 years.
- Analysis of the *MEN1* gene should be offered and extended to patients with atypical presentation of MEN-1, cases of suspected MEN-1 that do not meet clinical criteria (e.g. HPT association and thymic carcinoid tumour), young patients diagnosed with HPT for adenoma (<30 years) or parathyroid hyperplasia (<40 years) or multifocal pancreatic NETs.
- Patients with isolated gastrinoma may be investigated for association with primary HPT and family history of MEN-1, although the cost-effectiveness of genetic testing all cases, independently of age, is unknown.
- Patients with MEN-1 should be managed by an experienced multidisciplinary team.

### MEN-1: management of localised disease

Gastrinomas associated with MEN-1 are generally small, multiple, located in the duodenum and indolent, especially when lesions are <2 cm [114, 115]. The management of gastrinoma is mainly focused on the control of symptoms related to hypergastrinemia. The use of high doses of proton pumps inhibitors (e.g. omeprazole, 80–160 mg/day) is usually effective in controlling symptoms.

Most insulinomas are benign but must be found and operated regardless of their size. The goal of surgery is not disease control, but to control the clinical manifestations related to hyperinsulinemia [116].

#### Recommendations

- We recommend surgical exploration in cases of gastrinomas >2 cm, preserving pancreatic function if possible. The goal of surgery is to control disease progression and metastasis.
- We recommend surgery for all cases of localised insulinoma, with transoperative control of hypoglycemia (US with dosages of insulin and glucose).
- We recommend monitoring non-functioning pancreatic NETs <2 cm using conventional screening images at intervals of 6 months to 1 year [117, 118]. For tumours >2 cm, we recommend surgical intervention [113] [IVB].
- For patients with MEN-1-associated metastatic pancreatic NET, we prefer everolimus as the first option [119] [VC].

### MEN1: management of pituitary

#### Pituitary adenomas

The pituitary adenomas constitute about 15% of all intracranial tumours. The classification is morphological: microadenomas are <1 cm and macroadenomas are >1 cm. Both can be invasive, but pituitary tumours associated with MEN-1 tend to be more aggressive than sporadic forms. Adenomas can be secreting or not.

#### Recommendations

- The first choice of therapy for prolactinomas should include dopamine agonists, and cabergoline is the preferred medication [120, 121] [IA].
- Surgical treatment is recommended in cases of resistance or intolerance to dopamine agonists [121] [IVA].
- Temozolomide is indicated for rare cases of aggressive and resistant tumours [122, 125] [IIIB].
- Treatment with radiation therapy may be considered in tumours with local complications [121] [IVB].
- Repeated surgical resection can be performed in recurrent tumours [IVB].

### Acromegaly

Acromegaly is a rare disease, but with high morbidity and mortality rates. Surgery is the primary treatment for most cases. SA, in particular, dopaminergic agonists and GH receptor antagonists comprise the treatment for cases that are not controlled surgically or cases with restrictions to surgery. Radiation therapy has been less often used.

#### Recommendations

- Surgery is the first treatment option [122].
- We recommend the use of SA to treat symptoms and signs [IB]. Both octreotide and lanreotide are clinically equivalent [122].
- Pegvisomant can be used in SA-refractory cases [123, 124] [IB].

### Cushing’s disease

#### Recommendations

- Trans-sphenoidal surgery of the pituitary gland is the first choice of therapy [122] [IIA].
- We recommend drug therapy with ACTH modulators or blockers of adrenal synthesis of cortisol and/or radiation therapy for cases uncontrolled by surgery [122] [IVB].
- Bilateral adrenalectomy can be considered as an option if other therapies fail [122] [IIIC].

#### Clinically non-functioning pituitary tumours

- Pituitary surgery, usually by the trans-sphenoidal approach, is the treatment of choice [122] [IVA]
- Radiotherapy is indicated for recurrent or partially removed tumours [122] [IVA]

### Von Hippel–Lindau syndrome: clinical and molecular diagnosis

A germline mutation in the short arm of chromosome 3 causes the inhibition of von Hippel–Lindau protein, which results in the increased hypoxia-inducing factor leading to increased production of PDGF, a stimulator of epithelial and vascular cell growth, that promotes hypervascular cystic tumours, carcinomas including clear renal cells and pancreatic NET, with a penetrance of approximately 15%, as well as paragangliomas and pheochromocytomas [126].

#### Recommendations

- All patients with a clinical diagnosis of VHL syndrome, even without a family history, should carry out a molecular test [126] [IVA].
- We recommend screening VHL patients with usual clinical evaluation and annual imaging tests (abdominal US, CT or MRI) beginning at 20 years of age [VB]. To differentiate pancreatic cystadenoma and cysts with septate and thick content, we suggest octreoscans or PET/CT^68^Ga, if available.
- Genetic testing is also recommended for the first-degree and second-degree family members to identify asymptomatic carriers of VHL disease [VA].

### VHL: management of localised gastrointestinal disease

#### Recommendations

- We recommend radiological monitoring in cases of cysts in the pancreas. Surgery is indicated in exceptional cases for the compression of adjacent organs or development of NET.
- We recommend the approach used by Libutti *et al* [126] (Table 4) for patients with NETs of the pancreas [IVB].

### Distinct management for metastatic NETs associated with hereditary syndromes

Therapeutic management of GEP NET associated with hereditary syndromes should follow the same principles of sporadic NET. Only case reports are available to support management of these tumours.

#### Recommendations

- In general, we recommend the use of sporadic tumour treatment protocols [VA].
- We prefer sunitinib as the first choice in pancreatic NET associated with VHL syndrome [VC] and everolimus as the first choice in MEN-1-associated pancreatic NET [119][VC]

## Conclusion

The management of NETs is challenging and complex because of their vast heterogeneity. Thus, each case should be discussed and handled in a multidisciplinary environment. The development of national guidelines is an important roadmap for assertive diagnostic and therapeutic choices, not only based on scientific evidences but also adaptable to particular scenarios. In this Brazilian NET guideline, we constructed recommendations for diagnosis and treatment that differ from those found in current guidelines. We believe that our considerations are useful and can assist medical decisions for the best management of patients.

The incidence of NETs, although still low, has increased and will become more frequent in medical practice. Continuous efforts toward updated knowledge should be made, especially through collaborative groups. Working together can be more effective in standardising procedures, evaluating risks and benefits of available treatments, and optimising resources. Finally, clinical and translational research should be encouraged as it is essential in elucidating the carcinogenesis mechanisms of NETs and a key step in therapeutic advances.

## Figures and Tables

**Table 1:** Levels of Evidence—CDC Grading System (Adapted from [127]).

I: At least one randomised controlled trial (RCT) of good methodological quality or meta-analysis of well-designed RCT and without heterogeneity. II: Small RCTs or large RCTs with low methodological quality or meta-analysis of trials with high risk of bias. III: Prospective cohort studies. IV: Retrospective series or case-control studies. V: Case reports and expert opinion.

**Table 2:** Strength of Recommendations—Classification System CDC (Adapted from [127]).

A: Robust evidence of efficacy with significant clinical benefit; strongly recommended. B: Moderate evidence of efficacy, but with a limited or modest clinical benefit; usually recommended. C: Either insufficient evidence of efficacy or the benefit does not outweigh the risk/disadvantages (adverse events, costs, etc); optional. D: Evidence against the efficacy or risk may outweigh benefit; generally not recommended. E: Strong evidence against the efficacy or risk outweighs benefit; not recommended.

**Table 3:** Test Indication.

Test	Indication	Characteristics
Chromogranin A	General marker for NETs	Specificity and sensibility limitations
5-HIAA	Carcinoid syndrome, midgut tumour	Specific for carcinoid syndrome. Particular attention to potential false positive results
Gastrin	Gastric, pancreas and duodenum NETs	Useful for differentiation of stomach NET, indicated for pancreatic or duodenal gastrinoma
Secretin	Complementary to gastrin measurement (when levels are 200–1000 pg/mL)	Difficult to access
Insulin, pro-insulin, C Peptide after 72-h fasting	Insulinoma	Indicated when glycemic levels ≤nd mg/dl and concentration of insulin ≥6 µU/l. Hospitalisation required
Glucagon	Glucagonoma	After 8-h fasting

**Table 4:** Management of Pancreatic NET in von Hippel–Lindal Syndrome.

Size of pancreatic NET	Management
≤1 cm	Monitoring at 12-month intervals with CT and MRI
1–3 cm	Evaluate each case individually
>3 cm	Resection of symptomatic lesions (functioning or progressive lesions)

Resection: Injuries >2 cm in the head of the pancreas and >3 cm at the tail of the pancreas. Symptomatic lesions at any size. Abdominal intervention by any other tumour. Parenchyma-sparing surgery is the preferred surgical intervention [126, 128].
